# Enhancing esophageal repair with bioactive bilayer mesh containing FGF

**DOI:** 10.1038/s41598-021-98840-w

**Published:** 2021-09-28

**Authors:** Ozkan Cesur, Tugba Endogan Tanir, Pinar Celepli, Fatma Ozarslan, Sema Hucumenoglu, Adnan Karaibrahimoglu, Nesrin Hasirci

**Affiliations:** 1 Department of Pediatric Surgery, Ankara Training and Research Hospital, Health Sciences University, Hacettepe Mh., Ulucanlar Cd., No:89, Altındag, Ankara 06230 Turkey; 2 Central Laboratory, Middle East Technical University (METU), Dumlupinar Bulvari, Ankara, 06800 Turkey; 3 Department of Pathology, Ankara Training and Research Hospital, Health Sciences University, Hacettepe Mh., Ulucanlar Cd., No:89, Altındag, Ankara 06230 Turkey; 4 Department of Thoracic Surgery, Ankara Training and Research Hospital, Health Sciences University, Hacettepe Mh., Ulucanlar Cd., No:89, Altındag, Ankara 06230 Turkey; 5 Department of Biostatistics and Medical Informatics, Faculty of Medicine, Suleyman Demirel University, Isparta, 32260 Turkey; 6 Department of Chemistry, Middle East Technical University (METU), Dumlupinar Bulvari, Ankara, 06800 Turkey; 7 Center of Excellence in Biomaterials and Tissue Engineering (BIOMATEN), Dumlupinar Bulvari, Ankara, 06800 Turkey; 8 Tissue Engineering and Biomaterials Research Center, Near East University (NEU), Lefkosa, TRNC, Mersin 10 Turkey

**Keywords:** Health care, Medical research, Engineering

## Abstract

We aimed to prepare a bioactive and biodegradable bilayer mesh formed by fibroblast growth factor (FGF) loaded gelatin film layer, and poly ε-caprolactone (PCL) film layer, and to investigate its treatment efficacy on esophageal anastomosis. It is envisaged that the bioactive mesh in in vivo model would improve tissue healing in rats. The full thickness semicircular defects of 0.5 × 0.5 cm^2^ were created in anterior walls of abdominal esophagus. The control group had abdominal esophagus isolated with distal esophageal blunt dissection, and sham group had primary anastomosis. In the test groups, the defects were covered with bilayer polymeric meshes containing FGF (5 μg/2 cm^2^), or not. All rats were sacrificed for histopathology investigation after 7 or 28 days of operation. The groups are coded as FGF(−)-7th day, FGF(+)-7th day, and FGF(+)-28th day, based on their content and operation day. Highest burst pressures were obtained for FGF(+)-7th day, and FGF(+)-28th day groups (p < 0.005) and decreased inflammation grades were observed. Submucosal and muscular collagen deposition scores were markedly increased in these groups compared to sham and FGF(−)-7th day groups having no FGF (p = 0.002, p = 0.001, respectively). It was proved that FGF loaded bioactive bilayer mesh provided effective repair, reinforcement and tissue healing of esophageal defects.

## Introduction

Esophageal replacement studies come to the fore in invasive neoplastic diseases, congenital pathologies and transmural caustic injuries^1^. In general, primary anastomosis applied to repair congenital esophageal pathologies is not possible if long gap esophageal atresia is present^2^. After primary surgical repairment is applied in childhood esophageal pathologies, problems such as stenosis, leakage, infection and fistula recurrence may occur in the anastomotic area and may require a second surgery. The applicability of bioactive meshes to meet these difficulties is a highly researched area^3^. These scaffolds are used to regenerate tissue and maintain the function of organs^4^. This field of science is based on multidisciplinary studies with cellular biology, materials engineering, physiology and gene therapy, using engineering principles^5,6^. Preclinical animal experimental studies for esophageal repair, especially the use of small species such as murine species, provides robust statistical results and cost-effectiveness for determining regenerative medicine strategies^1^. The preparation of structures to replace the esophagus is based on the use of biological and decellularized or synthetic scaffolds^1^. The biological scaffold derived from extracellular matrix (ECM), that is present in all tissues and been essential for life, is very important for the treatment of wounds. ECM is a complex three-dimensional (3D) acellular fibrous structure that creates a protective and supportive dynamic mesh and reciprocal signaling platform for pragmatically acting cells at the wound area^7^. Hydrogels made of natural polymers such as collagen, alginate, chitosan or gelatin can be used to mimic the natural 3D network structure of ECM^8^. In our previous studies, we have shown that protein based-polymers such as collagen, gelatin and fibrin have the advantage of creating a suitable ECM environment and increasing cell proliferation and tissue regeneration^9–12^. Collagen, an important component of ECM, provides fibrogenesis and integrin-mediated mechanical attachment. It creates stiffness and porosity that allows the cells to migrate together. Cellular mobility and translocation ability are modulated by collagen^13^. Collagen is the most widely found protein in the body. Gelatin is a natural polymer obtained by controlled hydrolysis of collagen and contains glycine, proline, hydroxyproline and residues that aid cell adhesion and differentiation. It is attractive for tissue engineering applications because it is biodegradable, biocompatible and absorbable^14^.

Natural and synthetic polymers used for the preparation of scaffolds are used as supports for tissue regeneration and healing. Both types of materials have several advantages and disadvantages^15^. Synthetic scaffolds are widely used in esophageal repair works because they are easily obtained with reproducible and reliable processes, are xeno-free, and are low cost. It has been reported that there are difficulties to be overcome in the use of natural or synthetic meshes such as anastomotic leakage, local infection, graft failure and optimal graft disruption^16^. Synthetic scaffolds acted as a natural stent providing re-anastomosis, causing fibrosis in the esophagus. It has been reported that successful clinical results have not been obtained in the use of very soft and permeable scaffolds. Although many interesting achievements have been made about the proposed natural biomaterials for forming esophageal tissue, problems such as poor mechanical strength and rapid degradation have been reported in some vitro or vivo experiments. Adequate muscle regeneration and vascularization have not been observed in experimental use for esophageal repair of porcine dermal collagen scaffold as naturally produced mesh^17^. It is not easy to obtain a suitable structure that meets all the requirements for a successful scaffolding. In some studies, instead of a single-layer structure, bilayer (two-layer) systems have been developed as a combination of natural and synthetic polymers for tissue regeneration to take advantage of the required properties of both materials^15^. There is no consensus or comparative study on the superiority of natural or synthetic materials in esophageal repair in the literature^16^. There is not enough information about its long-term results^1^. Also, various growth factors (GFs) can be added to the construct to support tissue regeneration^18^. GFs can be added directly to polymeric matrices or loaded into micro or nanoparticles and then added to scaffold material^19–21^. GFs are signaling molecules that guide cell development by providing biochemical cues for stem cell proliferation, migration and differentiation^14^. Due to their effects on cell signaling pathways, they are effectively used in tissue treatments and tissue engineering applications^22–24^. GFs have a powerful effect on the tissue repair process. Fibroblast growth factor (FGF) stimulates the growth and differentiation of many cells from skeletal muscle cells to smooth muscle cells, including endothelial, chondrocyte, keratinocyte, melanocyte and glial cells^25^. It also acts as a potent angiogenic factor by stimulating reperfusion and angiogenesis. In esophageal repair studies, as in all anastomosis healing studies, it is aimed to increase submucosal collagen formation, which is the most important factor affecting wound healing^19,26^. In a study on FGF, it was shown that the use of exogen has a positive effect on esophageal wound healing^19^. Loading tissue-specific GFs and antibodies into biodegradable smart synthetic nanofibers can provide functional results by improving wound healing^27^. Intelligent matrices containing factors that promote tissue regeneration may allow for required cell seeding and obtain a standard in vivo biological response^28^. Poly ε-caprolactone (PCL) is one of the aliphatic polyesters and widely used in tissue engineering structures due to its biocompatibility, mechanical strength, suitability for modification and low cost^29–31^. PCL is one of the biocompatible polyesters that can be used in the production due to its neutral, natural and long-term biodegradability. As the PCL-based scaffold breaks down, it is replaced by new tissue, leaving no synthetic material behind. The decomposition products that emerge during their biological degradation are also noteworthy because they do not cause any harm to the body^31^. Replacing the hydrophobic nature of PCL with hydrophilic and bioactive molecules enhances the healing effect of the wound healing process^32^. PCL is prepared in various forms such as films, mats, nanofibers, nano/micro particles or meshes^31^. PCL is used with natural polymers such as gelatin to provide better cell adhesion and proliferation due to integrin binding the tripeptide *Arg-Gly-Asp (RGD)* motif^32,33^. Human esophageal epithelial cell proliferation was higher in PCL-gelatin nanofiber scaffold, and thus the PCL-gelatin nanofiber scaffolds were presented as potential candidates for regeneration of functional esophagus^34,35^. The main purpose of our study was to prepare a bilayer mesh consisting of fibroblast growth factor (FGF) loaded gelatin on the PCL layer in order to provide the desired tissue repair with healing cell epithelization for esophageal damages. Cell adhesion can be activated by growth factors (GFs) and protein-based gelatin, while PCL will provide mechanical strength to the mesh.

In esophagial repairs, no studies have been conducted on the effect of esophagial wound healing supported by a suitable mesh structure with growth factors (GFs) in its composition in esophageal repairs. In our study, it was aimed to investigate the healing effect of a bilayer mesh structure consisting of gelatin and poly ε-caprolactone (PCL), in which fibroblast growth factor (FGF) is loaded into the gelatin, which has a porous structure that mimics the extracellular matrix (ECM). It was aimed to show the positive effect on strengthening of mesh esophageal defects and healing of esophageal anastomoses. To our knowledge, our study is the first to use a bilayer mesh made of FGF for reinforcement of esophageal defects. There are studies FGF in a single layer gelatin film or using epithelial growth factor (EGF) as a local single dose for the treatment of esophageal defects^19,24^.

## Materials and methods

### Preparation of bilayer meshes

**Figure 1:**
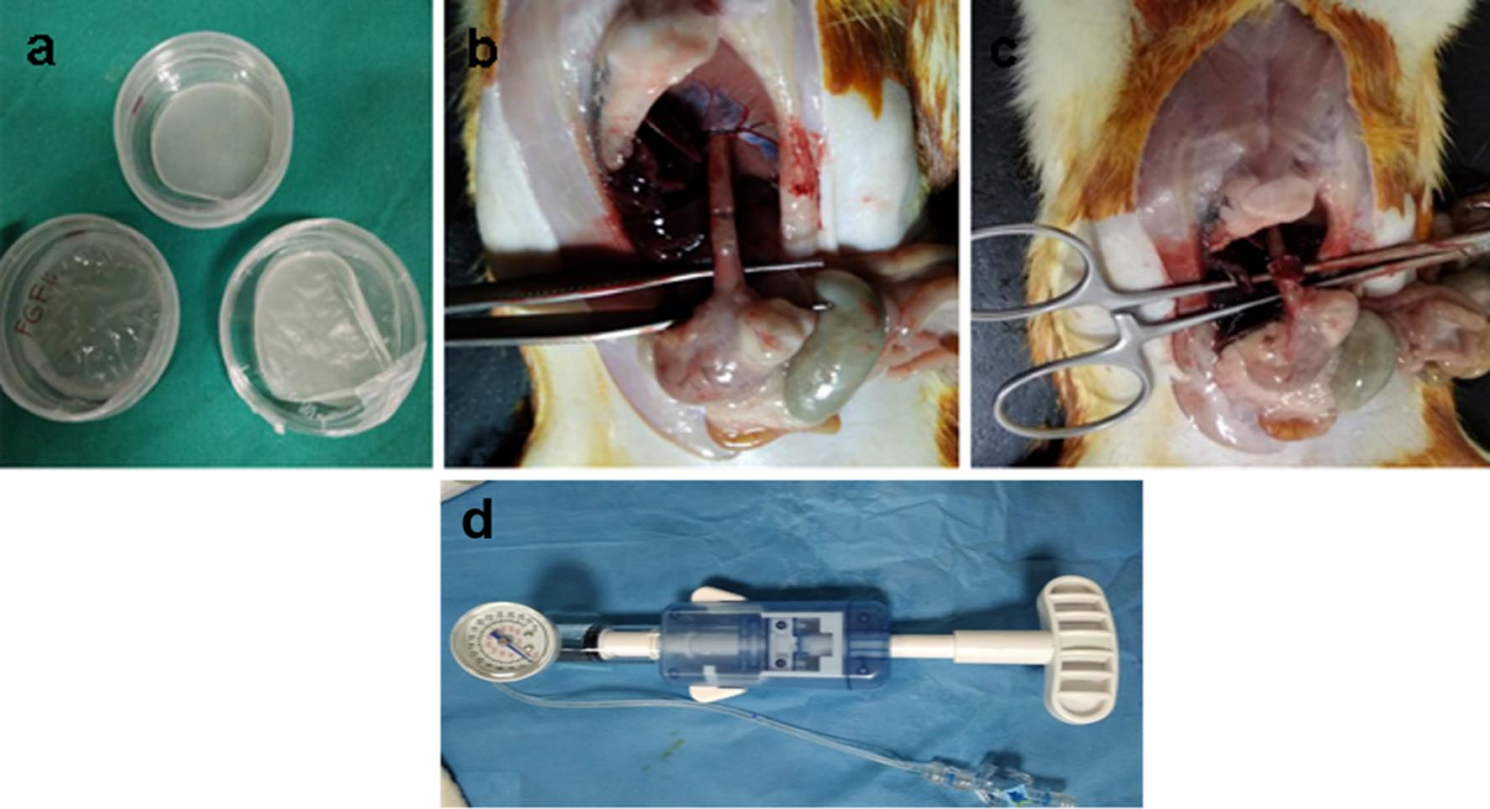
Design of study (**a**) bilayer meshes, (**b**) preparation of esophagial defect, (**c**) the mesh was placed as a patch over the esophageal opening, then the edges of the esophageal opening were secured with interrupted sutures, (**d**) syhngomanometer device.

Polymeric bioactive bilayer meshes made of poly ε-caprolactone (PCL) (Mn:80.000 Da, Aldrich, UK) film and gelatin (Scharlau, Spain) film were prepared. As a first layer, PCL films were prepared by solvent casting technique. For this purpose, 5% (w/v) PCL solution was prepared in a dichloromethane (Across) and poured into 10 mm diameter glass petri dishes as molds. Dry PCL films were obtained after the evaporation of the solvent, and films were immersed in 10% hexane diamine-isopropanol solution for 1 h at 37 °C for aminolization. These PCL films were washed with deionized water for 24 h at room temperature to remove the remaining free 1,6 hexane diamine. In a separate beaker, gelatin solutions were prepared with addition of FGF (Sigma). The effective dose of FGF was given as 120 ng per 100 g/d^19^. By taking this as a reference, gelatin films containing 2.5 µg FGF per cm^2^ of films were prepared. For this purpose, aqueous gelatin solution (5% w/v) was prepared in distilled water, glutaraldehyde (0.05% w/v) was added as crosslinker to stabilize gelatin. After mixing this solution for 1 min, FGF solution was added and the total solution was poured on to aminolyzed PCL films. Gelatin solution having no FGF was also prepared and added on PCL to compare the effect of FGF. The bilayer mesh structures were obtained after the films were dried at room temperature (Fig. 1). The ones having FGF and no FGF are coded as FGF(+) and FGF(−), respectively (Table 1).

**Table 1:** Contents of bilayer meshes applied to animals.

Bilayer mesh	Content
FGF(−)	Form from gelatin film and PCL film as second layer (has no FGF). Films of 1 × 2 cm^2^ were applied to rats
FGF(+)	Contains 2.5 μg FGF per cm^2^ of gelatin film and, PCL film as second layer. Films of 1 × 2 cm^2^ having 5 μg FGF were applied to rats

### Experimental animals

In the study, 34 male adult wistar-albino rats, the body weight ranging from 200 to 250 g, were provided by Husnu Sakal Experimental and Practice Center, Ankara Training and Research Hospital, Health Sciences University, Ankara, Turkey. Rats were kept in special cages at a standard room temperature (24 °C) in a 12 h light/dark circadian rhythms. Animals were fed with standard pellet feed and free access via city water. Institutional Ethical Approval was obtained from the Animal Experiments Local Ethics Committee**,** Ankara Health Research Application Center, Health Sciences University, Ankara, Turkey (Ethical approval number: 17/09/2019-0055).

### Study design

In the study, 34 male wistar-albino rats were randomly divided into five groups, having 6 rats in Control Group, and 7 rats in each of the other test Groups. After 12 h of fasting, 10 mg/kg xylazine (Rompun; Bayer AG, Leverkusen, Germany) and 50 mg/kg ketamine hydrochloride (Ketalar; Parke Davis, Eczacibasi, Istanbul, Turkey) were given to rats under anesthesia via intramuscular injection. All procedures were performed in sterile environment. Before the abdominal incision, a silastic 8 F orogastric feeding catheter (Bicakcilar, Turkey) was placed into the stomach via the oral route. Under aseptic and antiseptic conditions, 3 cm midline laparotomy was performed. The distal esophagus was visualized and mobilized for 4 cm length using blunt dissection. A full thickness semicircular defect of 0.5 × 0.5 cm^2^ was created via cutting in the anterior wall of the abdominal esophagus. The defect was either repaired with primary anastomosis without mesh, or with the prepared bilayer mesh using an interrupted absorbable suture of 6–0 polyglactin (Vicryl; Ethicon, USA) (Fig. 1). The Control Group had only distal esophageal blunt dissection, and was isolated. The Sham Group had primary anastomosis without mesh application. Bilayer mesh without FGF were applied to animals and these animals were sacrified after 7 days (FGF(-)-7th d Group) for examination. Bioactive bilayer meshes (2 cm^2^ having 5 μg FGF) were applied other two groups and these animals were sacrified after 7 days (FGF(+)-7th d Group), and 28 days (FGF(+)-28th d Group) for examination. The abdominal wall and skin were closed by layer with continuous 3–0 silk (Silk; Ethicon, USA) suture. For all group animals, before the abdominal incision closure, 5 mL of 0.9% NaCl was administered intraperitoneally. After the first 24 h, the rats were allowed free access to food and water. The Control, Sham, FGF(-)-7th d, FGF(+)-7th d and FGF(+)-28th d Groups animals all were sacrificed exsanguination under deep anesthesia at the end of experiments. Lack of movement, absence of heart beat, pupillary response to light and respiratory pattern were confirming death in all experimental animals. For investigation 4 cm distal esophagus including anastomotic site was resected, and bursting pressure, inflammation and collagen deposition values were determined by histopathological tests.

### Burst pressure measurement

Burst pressure was measured infusionally with indeflator system, which have pressure transducer, pressure channel, and sphingomanometry (BIG60 Inflation Device, Merit Medical System Inc., Utah, USA). After 4 cm distal esophagial segment included anastomotic area was resected, pressure channel catheters were placed within the proximal end. The distal end was tied with a 2/0 silk suture. Monitoring intraluminal pressure with this device, the point of leakage from the anastomosis occurred was recorded as the bursting pressure (Fig. 1).

### Histopathological examination of the esophagus

Tissue samples of 4 × 2 cm^2^ size of the esophagus obtained from rats were embedded in paraffin after gradual ethanol dehydration (50%, 75%, 96% and 100%, respectively) and xylene translucency following fixation in 10% formaldehyde solution for 2 days. 4 µm sections were taken with Leica RM 2125 RT microtome from paraffin-embedded tissues. Tissue sections were examined by staining with Hematoxylin&Eosin (H&E) and Mason Trichrome Stain. Histopathological examination was evaluated microscopically using OLYMPUS brand, BX51TF model, ×4, ×10, ×20, ×40 lenses. The esophageal collagen density, epithelialization and polymorphonuclear leukocytes (PMNL) were evaluated semiquantitatively with the histopathological scoring system using Abramov's Histologic Scoring System^36^ (Table 2).

**Table 2:** Description of the histopathological scores of the semi-quantitative evaluation.

Histological parameter	Criteria	Score
Submucosal collagen deposition	None	0
	Mild (submucosal collagen < muscularis mucosa thickness × 2)	+ 1
	Marked (submucosal collagen > muscularis mucosa thickness × 2)	+ 2
Muscular layer collagen deposition	None	0
	Mild (collagen deposition around the smooth muscles	+ 1
	Marked (collagen deposition around the smooth muscles and replacement of muscles with collagen)	+ 2
Epithelialization	Thickness of cut edges	0
	Migration of epithelial cells	1
	Moderate	2
	Bridging of the excision complete regeneration	3
PMNL, polymorphonuclear leukocyte	Minimum	0
	Mild	1
	Moderate	2
	Marked	3

### Statistical analysis

Statistical analysis of the study was performed with SPSS 20.0 Program (IBM Inc., IL, USA). Descriptive features were presented as frequency and percentage. The differences of histopathological evaluations according to the study groups were determined by the Chi-Square Analysis Method. P < 0.05 value was considered statistically significant (Table 3).

**Table 3:** Results of the semi-quantitative histopathological evaluation and bursting pressure, presented by its mean and standard deviation (mean ± sd).

Group	Submucosal collagen deposition	Muscular layer collagen deposition	Epithelisation	PMNL	Bursting pressure (mmHG)
Sham	0.85 ± 069	1.14 ± 0.37	0.42 ± 0.53	2.85 ± 0.37	20.84 ± 2.98
FGF(−)-7th day	0.42 ± 053	0.71 ± 048	1.28 ± 0.75	2.57 ± 0.53	29.94 ± 4.55
FGF(+)-7th day	1.6 ± 06	1.75 ± 0.46	2.12 ± 0.83	1.25 ± 0.46	53.52 ± 1.85
FGF(+)-28th day	2 ± 0*	2 ± 0*	2.6 ± 0.89*	0.6 ± 0.54*	60.15 ± 7.46*

M, mean, SD, standard-deviation.

*Chi-square test, significant at 0.05 level, (p < 0.05).

### Ethical statements

All authors declare that this manuscirpt is orginal, has not been published before and is not currently being considere for publication elsewhere.

### Informed consent

The care and use of laboratory animals is approved by the Animal Experiments Local Ethics Committee, Ankara Health Research Application Center, Health Sciences University, Ankara, Turkey (Ethical approval number: 17/09/2019-0055). According to the study, 34 male adult wistar-albino rats, weighing between 200 and 250 g, were in good health and had no diseases. The procedures performed in studies involving animals followed the ethical guidelines outlined in the Declaration of Helsinki from 1964.

Husnu Sakal Experimental and Practice Center, Ankara Training and Research Hospital, Health Sciences University, Ankara, Turkey provided the animals. All of the animals were addressed and used in accordance with the Animal Research: Reporting of in Vivo Experiments (ARRIVE) quality assessment criteria including ethical statement, study design, experimental procedure, experimental animals, housing and husbandry, simple size, allocating animals to experimental groups, experimental outcomes, statistical methods, baseline data, number of analysed, outcomes and estimation and adverse events.

## Results

In our study, on post procedural course animals were survived in all groups. For collecting of the post operative information, weight loss, analgesics administrated, wound healing, activity and feeding was recorded at the animal’s cage card.

When groups were sacrified, local abscess formation was observed macroscopically in Sham Group and FGF(−) Group, but not in FGF(+) Groups.

**Figure 2:**
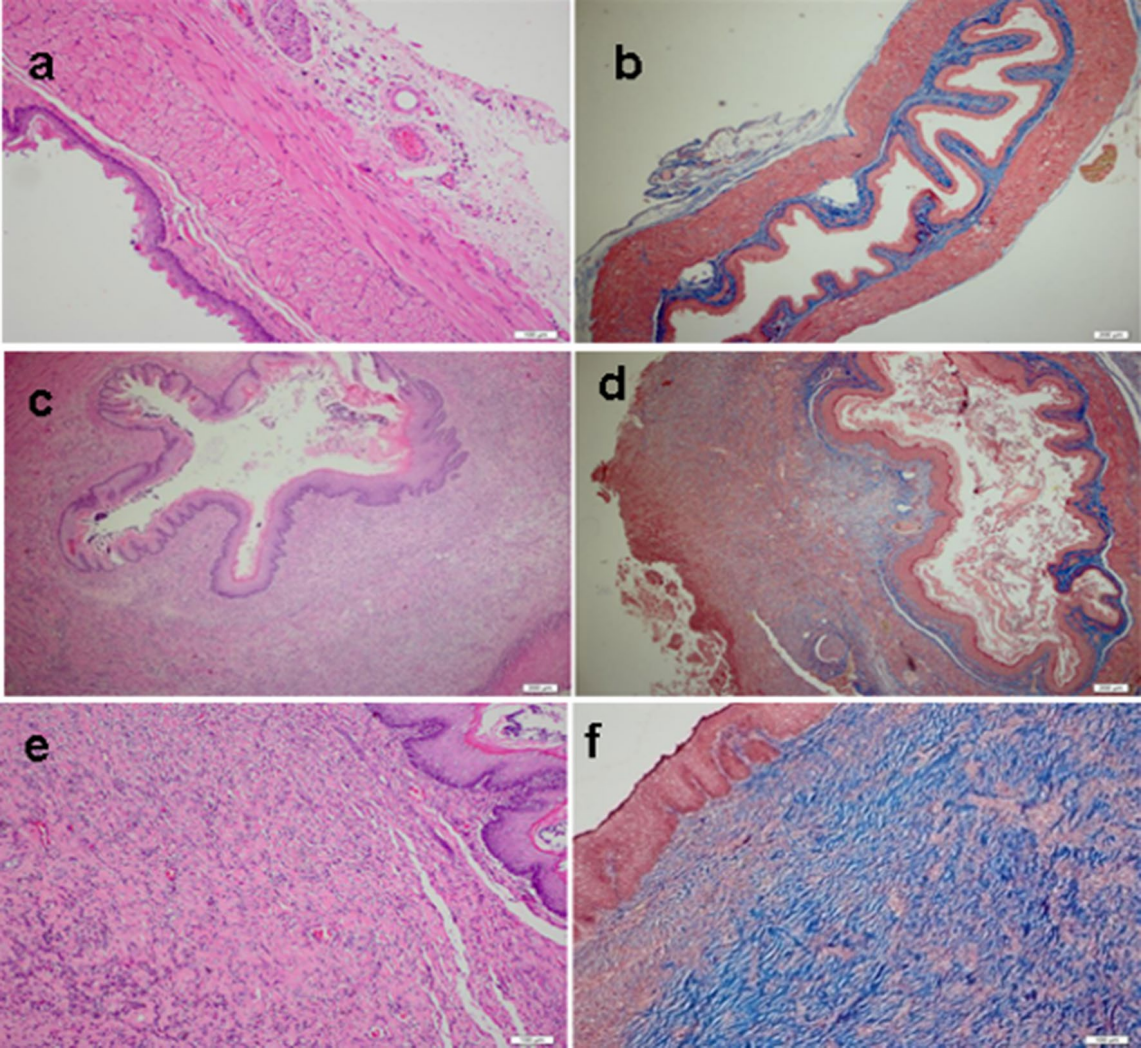
Mean histopathological pictogrammes of the groups studied. (**a**, **b**) Esophageal mucosa belonging to the control group. (**a**) There is normal archetype preserved esophageal full-thickness mucosa sample containing lamina propria and muscularis propria consisting of compact connective tissue observed under the surface ceratinized squamous epithelium, in the Hematoxylin&Eosin (H&E), × 100). (**b**) There is no collagen increase in the submucosal and muscular layers, in the Mason Trichrome Stain, × 100. (**c**, **d**) Esophageal mucosa belonging to the groups of sham and FGF(−). For the Groups of Sham and FGF(−) not received FGF, there are noteworthy epithelial degeneration, intense inflammation in the submucosa and muscular layer, and mildly increased collagen, in the Mason Trichrome Stain, × 100 and Hematoxylin&Eosin (H&E), × 100. (**e**, **f**) Esophageal mucosa belonging to the groups given FGF. (**e**) In the surface epithelium, there is marked regeneration as well as the presence of significantly increased collagen, which is replaced by the submucosal and muscular layer. There is a moderate decrease in the inflammatory cell density observed between the collagen bundles on the 7th day and a significant decrease on the 28th day, in the Hematoxylin&Eosin (H&E), × 100. (**f**) There is significantly increased collagen (blue color) between the submucosal and muscular layers observed, in the Mason Trichrome Stain, × 100.

**Figure 3:**
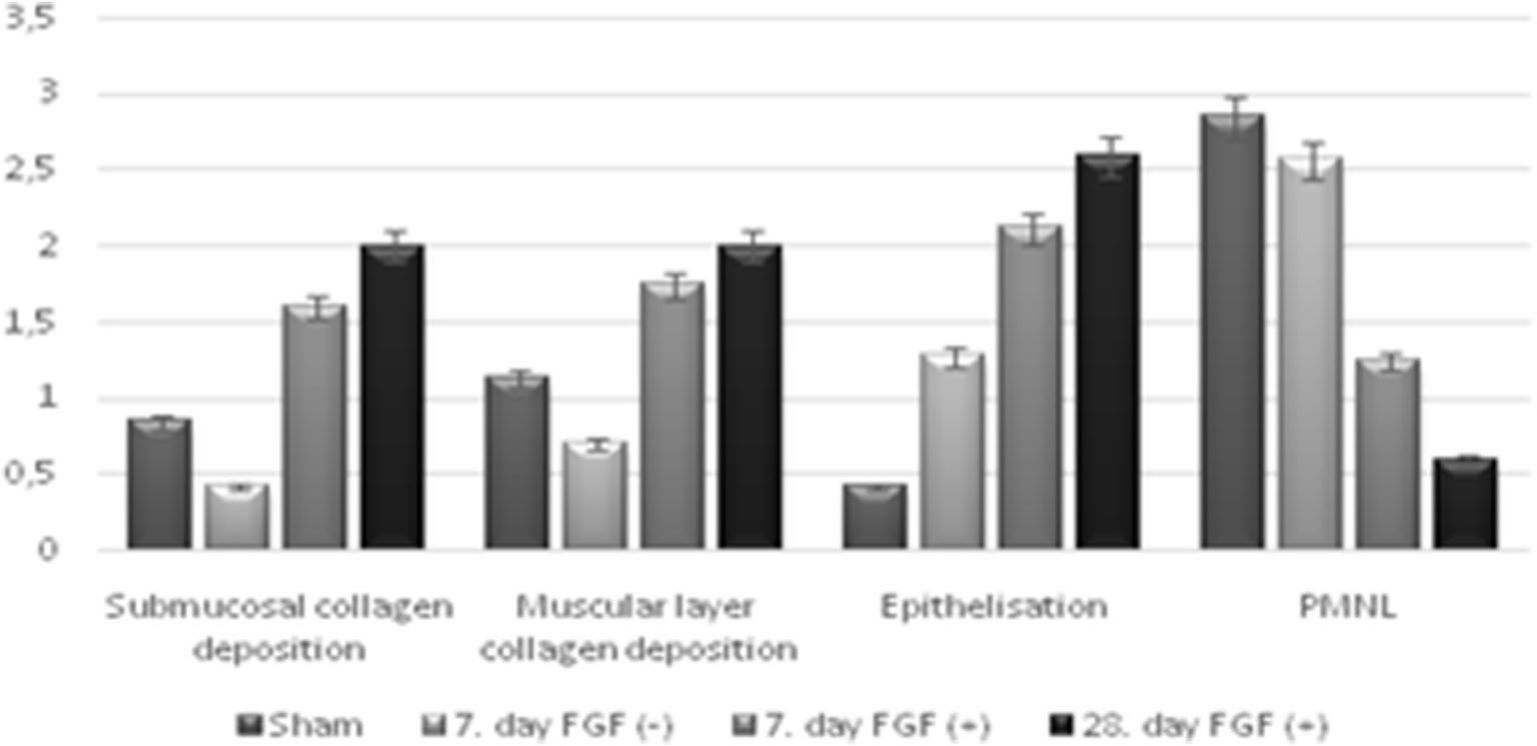
Mean histopathological scores according to groups.

No histological changes were observed in Control Group representing the normal esophagus histological findings. Sham Group and FGF(−) Group showed that epithelial regeneration did not occur. However, intense inflammation in the submucosal and muscular layer, and mildly increased collagen content were determined (Figs. 2, 3).

In bioactive groups having FGF, there was the surface epithelium regeneration as well as significantly increased collagen, which was replaced by the submucosal and muscular layer. Moderate decrease on the 7th day and significant decrease on the 28th day were observed in the inflammatory cell density between the collagen bundles. Significantly increased collagen (blue color) between the submucosal and muscular layers was observed with Mason Trichrome Stained tissues. When FGF(+) Groups were compared with the FGF(−) Group and Sham Group, the epithelial regeneration and collagen deposition in layers were significantly accelarated and inflammatory cells were decreased (p<0.05) in 7th and 28th days (Table 3, Fig. 3).

**Figure 4:**
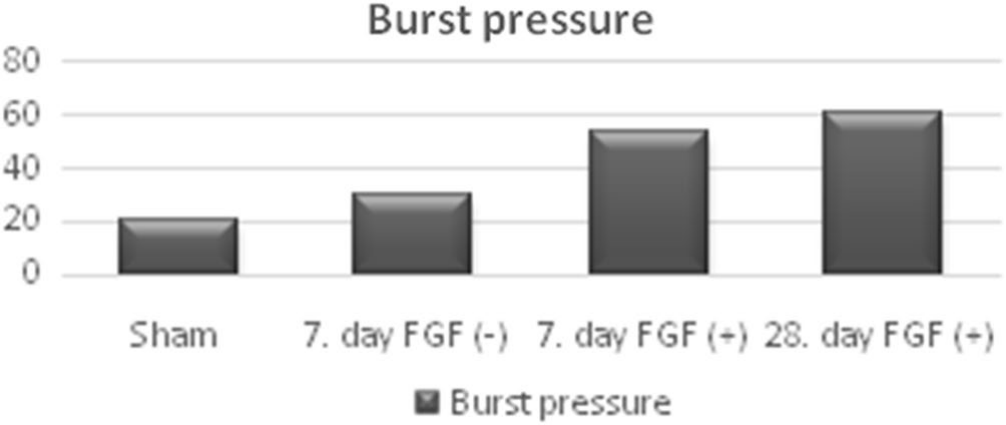
Differences of mean bursting pressure in the groups.

The burst pressures of the esophagial segments demonstrated variations. The ones which treated with FGF containing bioactive meshes (FGF(+) Groups) showed statistically significant higher burst pressure values with respect to the Sham and FGF(−) Groups (Table 3, Fig. 4).

## Discussion

Tissue regeneration technologically combines artificial scaffolds, cell-matrix and growth factors (GFs) to provide double or triple matrix products that can be applied in tissue repair and remodeling. It has been confirmed by Zhu et al. that these scaffolds can provide functional esophagus substitution well enough to support the growth of pig esophageal cells such as epithelial, fibroblast and muscle cells^37^. The effects of growth factors (GFs) on wound healing have been the subject of many studies^9,20,24^. Of these, fibroblast growth factor (FGF) is characterized as a strong mitogen for fibroblasts, capillary endothelial cells and mesenchymal cells. Therefore, it has been suggested that FGF plays a key role in accelerating wound healing, activating fibroblasts and inducing neovascularization^38^. With the electrospinning technique, it is possible to design double layer matrices with nanofibers consisting of poly ε-caprolactone (PCL)/poly-L-lactic acid (PLLA) for the lower layer and PCL/gelatin for the upper layer and gelatin microspheres are included in the middle of the two layers for controlled growth factor transmission^39^. Also it was reported that the sandwich scaffolding system have anti-leak and cell-binding support properties^40^.

In our study, it was aimed that it is the first study in terms of evaluating the short and long term results of mesh and growth factor (GF) that will create a barrier for sealing in esophageal anastomoses and increase cell migration and proliferation. When poly-ε-caprolactone (PCL) nanofiber layer was used in rabbit esophageal repair, an improvement in both epithelial and smooth muscle cells was provided. When polyvinylidene fluoride (PVDF) and absorbable vicryl surgical layer were used, morbidity and mortality rates were higher compared to the PCL nanofiber layer^41,42^.

In esophageal repair studies using poly-e-caprolactone (PCL) material, it has been reported that epithelial and smooth muscle cells can be observed within a postoperative month. Investigating the efficacy of random PCL and PCL-gelatin nanofibrous scaffold using human esophageal epithelial cells, Kuppan et al. found that epithelial cells were completely covered with epithelial cells after showing rapid adhesion and spread on PCL and PCL-gelatin nanofibrous scaffolds. They reported that PCL-based scaffolds help epithelial cell adhesion, showing the characteristics of living and cobblestone, enabling these cells to grow and proliferate^35^.

There is vivo model study in the literature that Senyucel et al. reported that local and sustained release of fibroblast growth factor (FGF) increased wound healing in esophageal anastomoses^19^. In this study, 24 male wistar-albino rats were used by dividing the animals into 3 groups. They performed abdominal esophageal resection and then end-to-end anastomosis to a 1 cm segment in all groups. In the control group, they performed a primary anastomosis, one group made an FGF-free gelatin film, the other group anastomosis supported with FGF and gelatin film. In all groups sacrificed on the postoperative 7th day, bursting pressures and histopathologically collagen deposition and tissue hydroxyproline concentrations in the anastomosis area were examined. They reported that local and continuous FGF release significantly increased burst pressure, and tissue OHP level in the anastomosis line, and the submucosal and muscle collagen concentration was higher than control groups. They showed that FGF provides remodeling by increasing fibroblastic proliferation and collagen maturation. They also reported an increase in epithelial healing. They observed that this epithelial cell restitution is highly advantageous in increasing water/airtightness in esophageal wound healing. They also suggested that fibroblast growth factor (FGF) may induce recovery by inducing angiogenesis induction and esophageal anastomosis, but more studies are needed to demonstrate the effect of neovascularization due to immunohistochemistry and capillary density. Although the increased collagen accumulation effect in the anastomosis region was seen to increase the healing effect in the short term, they could not give any results on whether it could lead to potential stenosis in the long term.

In our study, it was seen that until the 28th day when the rats were sacrified, no feeding difficulties were observed, and also no fibrosis was detected in the immunopathological examination. However, since our experimental animals are very small, there is no physical examination for possible stenosis. But, when the esophagus was catheterized and excised before sacrification, no stenosis was detected.

There is another vivo model study using epithelial growth factor (EGF) as a growth factor (GF) in esophageal repair. Adam et al. suggested that by applying a local single dose (100 pictogram) EGF to the anastomosis line, EGF can benefit from cell proliferation and migration of any epithelial cell during the esophageal wound healing process^43^. They did not find any difference in inflammatory scoring between the groups. However, they detected that collagen accumulation was increased in the esophageal layers in the group treated with EGF, which was sacrificed after 21 days, and EGF strengthened the anastomosis line. They reported that long-term effects of fibroblast growth factor (FGF)-gelatin film application with the local sustained release were not reported. Unlike the study of Senyucel et al., they reported that single-dose EGF application did not cause fibrosis on the 21st day of stenosis. Therefore, they reported that the application of the GF, which is planned to be applied to the esophagus anastomosis line, will not cause problems such as stenosis and leakage in esophageal wound healing.

The strength of our study that when the esophageal anastomosis was performed with fibroblast growth factor (FGF) loaded poly-e-caprolactone (PCL)-gelatin mesh, we not only showed that the esophagus healed with fibrosis in the 28-day long-term, but also that in the FGF unloaded PCL-gelatin group, PCL-gelatin played a role like ECM, supporting the esophageal anastomosis line and contributing positively to wound healing. Besides, according to the results obtained in our PCL-gelatin experimental Group unloaded FGF, we found that the PCL-gelatin bilayer mesh played an extracellular matrix (ECM) role in the esophageal anastomosis line and positively contributed to wound healing. According to epithelial growth factor (EGF), the effect of FGF on acute and chronic wound healing has been observed promising.

In conclusion, it has been proved that it is possible to strengthen the anastomotic line by repairing the esophageal defect using a double-layer mesh design based gelatin and poly ε-caprolactone (PCL) loaded fibroblast growth factor (FGF).

**Figure 5:**
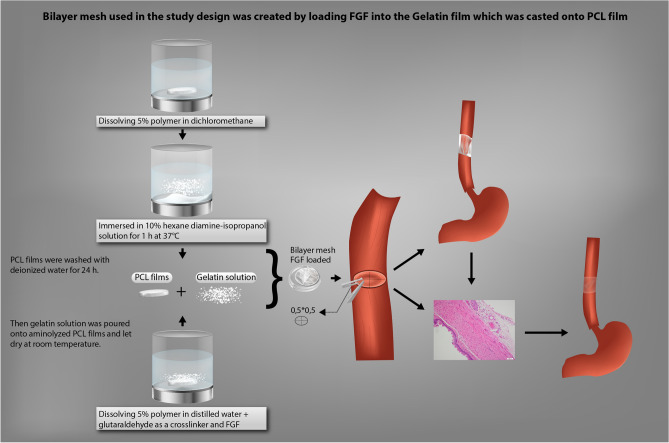
Bilayer mesh used in the study design was created by loading FGF into the Gelatin film which was casted onto PCL film showing preparation of bilayer meshes; PCL films were prepared by dissolving 5% polymer in dichloromethane. Dried PCL films were immersed in 10% hexane diamine-isopropanol solution for 1 h at 37 °C for aminolization. PCL films were washed with deionized water for 24 h. Gelatin solution was prepared by dissolving 5% polymer in distilled water and with addition of glutaraldehyde as crosslinker, and FGF. Gelatin solution was poured onto aminolyzed PCL films and let dry at room temperature. A full thickness semicircular defect of 0.5 × 0.5 cm^2^ was created via cutting in the anterior wall of the abdominal esophagus. The defect was repaired with primary anastomosis with the prepared bilayer mesh, using interrupted sutures.

In our study, we experimentally evaluated the therapeutic effect of esophageal wound healing on the healing of esophageal anastomoses using of the bilayer mesh design based fibroblast growth factor (FGF) enhanced with gelatin and poly ε-caprolactone (PCL), in rat esophagus (Fig. 5). The results achieved showed that using of this two-layer mesh based FGF enhanced with gelatin and PCL in rate esophagus healing has made a difference between the 7th and 28th. It was observed that the decrease in inflammation started on the 7th day was resulted in complete decrease in inflammatory cells, significant increase in collagen formation and completion of epithelization on the 28th day.

As demonstrated by these results obtained in our study, in esophageal tissue regeneration growth factors have an important role. Presence of FGF added bioactivity to the meshes which were prepared from gelatin (that mimic natural extracellular matrix, ECM), and poly-ε-caprolactone (PCL) (which have tissue compatibility and has strength advantages), and significantly affected epithelial regeneration and collagen accumulation.

It is mentioned that all available findings will be considered as a preliminary report. Finally, to achieve full scientific validity, using other research methods and advanced techniques (tube mesh with 3D software) and trying in larger animals such as pigs will fully clarify whether it is recommended for clinical use.
